# Increased autophagy leads to decreased apoptosis during β-thalassaemic mouse and patient erythropoiesis

**DOI:** 10.1038/s41598-022-21249-6

**Published:** 2022-11-03

**Authors:** Pornthip Chaichompoo, Ramaneeya Nithipongvanitch, Wasinee Kheansaard, Alisa Tubsuwan, Kanitta Srinoun, Jim Vadolas, Suthat Fucharoen, Duncan R. Smith, Pranee Winichagoon, Saovaros Svasti

**Affiliations:** 1 Department of Pathobiology, Faculty of Science, Mahidol University, Bangkok, Thailand; 2 Thalassemia Research Center, Institute of Molecular Biosciences, Mahidol University, Nakhon Pathom, 73170 Thailand; 3 Department of Clinical Microscopy, Faculty of Medical Technology, Mahidol University, Bangkok, Thailand; 4 Stem Cell Research Group, Institute of Molecular Biosciences, Mahidol University, Nakhon Pathom, Thailand; 5 Faculty of Medical Technology, Prince of Songkla University, Songkhla, Thailand; 6 Centre for Cancer Research, Hudson Institute of Medical Research, Melbourne, Australia; 7 Department of Molecular and Translational Science, Monash University, Melbourne, Australia; 8 Molecular Pathology Laboratory, Institute of Molecular Biosciences, Mahidol University, Nakhon Pathom, Thailand; 9 Department of Biochemistry, Faculty of Science, Mahidol University, Bangkok, Thailand

**Keywords:** Anaemia, Molecular medicine, Medical research, Paediatric research, Clinical genetics

## Abstract

β-Thalassaemia results from defects in β-globin chain production, leading to ineffective erythropoiesis and subsequently to severe anaemia and other complications. Apoptosis and autophagy are the main pathways that regulate the balance between cell survival and cell death in response to diverse cellular stresses. Herein, the death of erythroid lineage cells in the bone marrow from both β^IVS2-654^-thalassaemic mice and β-thalassaemia/HbE patients was investigated. Phosphatidylserine (PS)-bearing basophilic erythroblasts and polychromatophilic erythroblasts were significantly increased in β-thalassaemia as compared to controls. However, the activation of caspase 8, caspase 9 and caspase 3 was minimal and not different from control in both murine and human thalassaemic erythroblasts. Interestingly, bone marrow erythroblasts from both β-thalassaemic mice and β-thalassaemia/HbE patients had significantly increased autophagy as shown by increased autophagosomes and increased co-localization between LC3 and LAMP-1. Inhibition of autophagy by chloroquine caused significantly increased erythroblast apoptosis. We have demonstrated increased autophagy which led to minimal apoptosis in β-thalassaemic erythroblasts. However, increased PS exposure occurring through other mechanisms in thalassaemic erythroblasts might cause rapid phagocytic removal by macrophages and consequently ineffective erythropoiesis in β-thalassaemia.

## Introduction

Ineffective erythropoiesis causes severe anaemia in β-thalassaemia^1^. Erythrokinetic and ferrokinetic analyses have shown clear evidence that the bone marrow is the main site of erythroid cell death, with as many as 65% of erythroid precursors dying in the marrow^2^. Bone marrow from β-thalassaemia patients show marked erythroid hyperplasia, with an increased percentage of basophilic erythroblasts and polychromatophilic erythroblasts but with a decreased percentage of orthochromic erythroblasts^3–5^. Increased phosphatidylserine (PS)-bearing erythroid cells in the bone marrow from β-thalassaemia patients has been reported^2,4^. Accelerated apoptosis of β-thalassaemia erythroblasts has been demonstrated by increased PS exposure, DNA laddering and Hoechst 33342 positive staining in both ex vivo and in vitro culture systems of bone marrow erythroblasts from β-thalassaemia patients^2,4,5^. Apoptosis at the polychromatophilic erythroblast stage was also demonstrated in cell culture of erythroid progenitor cells obtained from β-thalassaemia bone marrow through annexin V/propidium iodide staining and TUNEL assay^4^. However, a low number of apoptotic erythroblasts was observed when compared with the degree predicted by ferrokinetic studies, suggesting that the ineffective erythropoiesis in thalassaemia may be caused by other mechanisms. In addition, decreased erythroblast differentiation, which may exacerbate ineffective erythropoiesis, has been observed in β-thalassaemic mice^6^. Nevertheless, other cell death mechanisms or stress response mechanisms may also play roles in the ineffective erythropoiesis occurring in β-thalassaemia.

Apoptosis is triggered by two major pathways, the extrinsic death receptor pathway or the intrinsic mitochondrial pathway^7^. The intrinsic apoptotic pathway can be initiated by a variety of intracellular stimuli including growth factor deprivation, DNA damage and oxidative stress, which cause mitochondria to release cytochrome c into the cytosol, where it combines with apoptotic protease activating factor 1 (Apaf-1) and activates the apoptotic initiator caspase, caspase 9, which in turn cleaves and activates the effector caspases, caspase 7 and 3. The extrinsic pathway of apoptosis is initiated by the interaction of a death ligand and its receptor such as Fas and Fas ligand (FasL). These proteins induce the formation of signaling complexes that lead to activation of the initiator caspase, caspase 8, and consequently triggering the activation of the effector caspases 3 and 7.

Autophagy has been shown to be an important process of many physiological conditions including cell survival, differentiation, development and homeostasis^8^. Under certain conditions, autophagy constitutes a stress adaptation that suppresses cell death and apoptosis, while under other cellular conditions it can constitute an alternative form of cell death. Several studies have elucidated the crosstalk between apoptosis and autophagy showing how complex this relationship is, and how critical it is for the overall fate of the cell^9–11^. Autophagy constitutes a cytoprotective response activated by cells in an attempt to cope with stress. However, if the stress is high and the induced injury irreversible, autophagy can turn into a cell death mechanism and substitute for apoptosis. Autophagy appears to be responsible for the death of apoptosis-deficient cancer cells in response to chemotherapeutic agents^12,13^. Moreover, mouse embryonic fibroblasts derived from mice that lack the functional apoptotic modulators BAX and BAK are resistant to apoptosis and die by autophagic cell death when treated with etoposide^14^.

In the erythroid lineage, autophagy is an important process in erythroid maturation including for mitochondrial clearance at the stage of terminal erythroid differentiation^15,16^. While autophagy is required for the maturation of erythroid cells, its role or contribution to disease, particularly in β-thalassaemia, has received little attention, although increased autophagy was observed during erythropoiesis of CD34^+^ cells derived from β-thalassaemia/HbE patients^17^.

Herein, apoptosis and autophagy in bone marrow erythroblasts from β^IVS2-654^-thalassaemic mice and β-thalassaemia/HbE patients were investigated. Elevated PS-bearing thalassaemia erythroblasts were observed. Interestingly, while the apoptotic response was not pronounced, evidence of enhanced autophagy at the early stage of erythroid differentiation was seen in bone marrow from both β-thalassaemic mice and β-thalassaemia/HbE patients. Inhibition of autophagy led to increased apoptosis and PS exposure, suggesting that autophagy is a protective mechanism in β-thalassaemia erythroblasts. Importantly, our results draw attention to the role that autophagy plays in ineffective erythropoiesis in β-thalassaemia.

## Results

### β-Thalassaemic mice have elevated PS-bearing basophilic erythroblasts but not increased apoptotic cells

**Figure 1:**
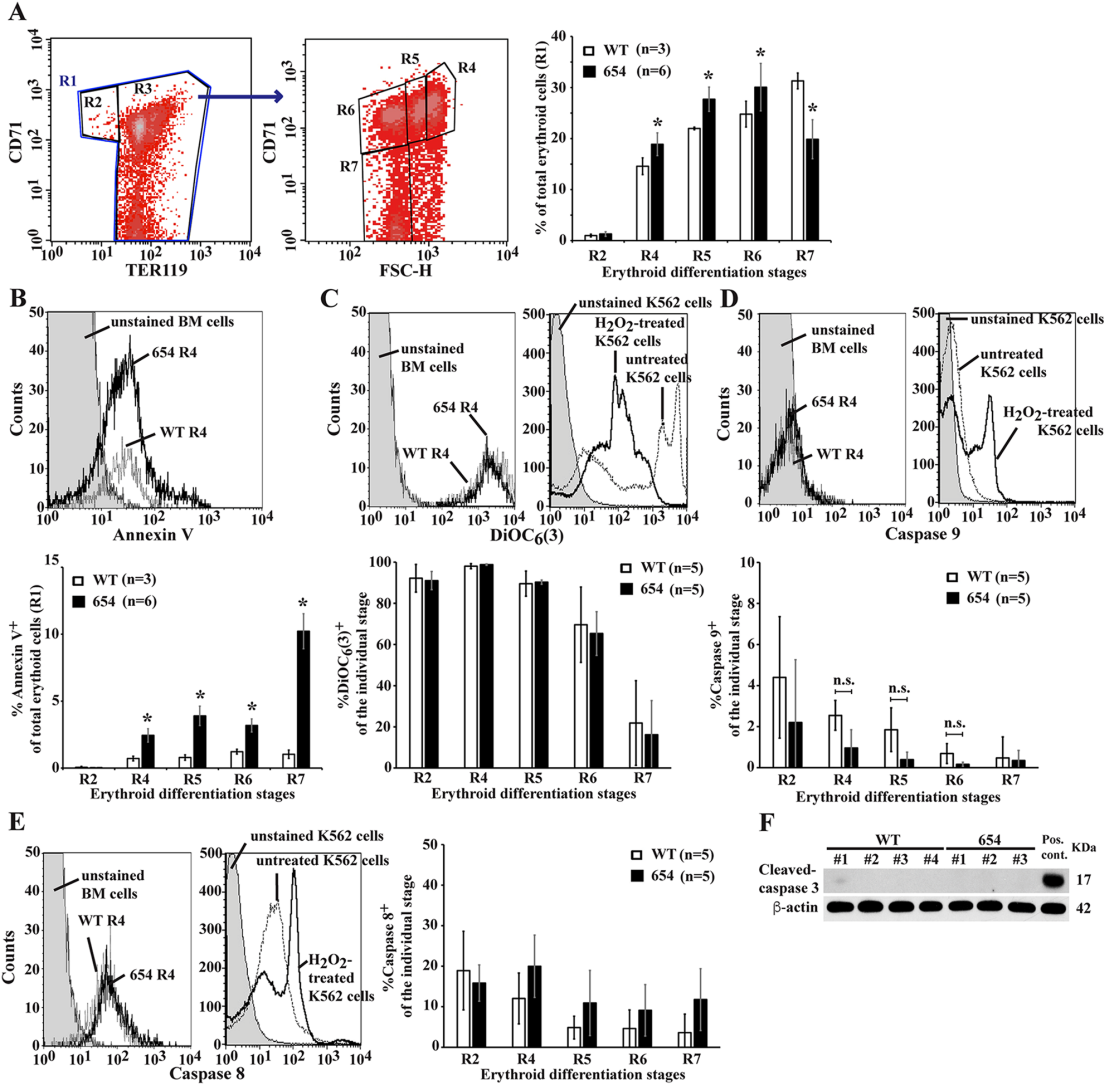
Elevated PS-bearing basophilic erythroblasts with no increased apoptosis in β-thalassaemic mice. Apoptotic markers were examined in bone marrow erythroid cells from β-thalassaemic mice (654) and wild type mice (WT). (**A**–**E**) Whole bone marrow samples were analysed for apoptotic markers in erythroid cells using flow cytometry. (**A**) Definition of flow cytometric erythroblast subsets. Bone marrow cells labeled with monoclonal antibodies specific to TER119 and CD71 and further analysed with respect to their forward scatter (FSC-H) (Supplementary Table 1). TER119^+^ erythroid cells in the R1 region were divided into two subpopulations TER119^dim^CD71^+^ erythroblasts (R2 region, proerythroblasts as majority as demonstrated in previous study^18^) and TER119^high^ erythroid cells (R3 region). Subsequently the R3 region was divided into a R4 region (basophilic erythroblasts as the majority cell type), a R5 region (polychromatophilic erythroblasts as the majority cell type), a R6 region (orthochromic erythroblasts and reticulocytes as the majority cell types) and an R7 region (red blood cells as the majority cell type) using CD71/FSC-H. The percentages of erythroid differentiation were analysed using a BD FACSCalibur flow cytometer and CellQuest Pro™ software (BD Biosciences). Percentages of (**B**) PS exposure (annexin V^+^ cells), (**C**) mitochondrial transmembrane potential (DiOC_6_(3)^+^ cells), (**D**) activated caspase 9, measured by Red-LEHD-FMK staining in living cells, and (**E**) activated caspase 8, measured by FITC-IETD-FMK staining in living cells, in each erythroblast subpopulation were examined (Supplementary Figs. S1 and S2). Unstained murine bone marrow cells were examined as a negative control. Hydrogen peroxide (H_2_O_2_)-treated K562 erythroleukemic cells were used as a positive control. Data are presented as mean ± S.D. *Significant difference when compared to wild type mice at *P* < 0.05, using Mann–Whitney U test. (**F**) Western blot analysis of cleaved caspase 3 in CD45^-^CD71^+^ bone marrow erythroblasts (Supplementary Fig. S3). The purity of CD45^-^CD71^+^ bone marrow erythroblasts was 73–91% as measured by flow cytometry. Cisplatin-treated CD45^-^CD71^+^ bone marrow erythroblasts were used as a positive control.

Apoptosis of β-thalassaemic mice erythroblasts was first determined by flow cytometry. Erythroid subpopulations were defined using CD71/TER119/FSC-H parameters in accordance with a previous study^18^. First, TER119 and CD71 staining identify two populations, the R2 region (CD71^+^/TER119^dim^), in which the majority cell populations were proerythroblasts and basophilic erythroblasts, and the R3 region (TER119^bright^), which contains a mixed population of erythroblasts that was further delineated by CD71/FSC-H staining into the R4 to R7 regions (Fig. 1A). The R4 region consists of basophilic erythroblasts as the majority population with proerythroblasts and polychromatophilic erythroblasts as minority populations. The R5 region consists of polychromatophilic erythroblasts as the majority population, while proerythroblasts, basophilic erythroblasts and orthochromic erythroblasts are minority populations. The R6 region consists of orthochromic erythroblasts and reticulocytes as the majority with polychromatophilic erythroblasts as the minority. Finally, the R7 region consists of mature red blood cells as the majority with reticulocytes as a minority^18^.

Bone marrow of β-thalassaemic mice have increased percentages of cells in the R4 to R6 regions, with a decreased percentage of cells in the R7 region as compared to bone marrow from wild type mice (Fig. 1A). Apoptosis of β-thalassaemic mice erythroblasts was first determined by staining with annexin V. A statistically significant increase in PS-bearing erythroid cells from the R2 to R7 regions (basophilic erythroblasts through to mature red blood cells) in murine β**-**thalassaemic bone marrow was observed as compared to cells from wild type mice (*P* < 0.05) (Fig. 1B, Supplementary Figs. S1 and S2). Although the ability of annexin V to bind PS with high affinity is a useful tool, it is not a definitive measurement of apoptosis. Observation of PS exposure on outer leaflet of cell membrane alone cannot discriminate between whether cells are undergoing apoptosis, or whether partial loss of phospholipid membrane asymmetry resulted from other factors. Therefore, other apoptotic markers of both the death receptor pathway and the mitochondrial apoptosis pathway were examined. Increased reactive oxygen species (ROS) in β-thalassaemia erythroblasts might cause mitochondrial damage and trigger the mitochondrial apoptosis pathway. However, the number of cells that had lost mitochondrial transmembrane potential and showed caspase 9 activation were minimal and not significantly different between β-thalassaemic and wild type mice (Fig. 1C, D, Supplementary Figs. S1 and S2). In addition, the initiator caspase of the death receptor pathway, caspase 8, was also minimally activated, but not different between the groups (Fig. 1E, Supplementary Figs. S1 and S2). The cleavage of caspase 3 was not detected in CD45^-^CD71^+^ bone marrow erythroblasts from either strain of mice (Fig. 1F). As activation of caspase 8, caspase 9 and caspase 3 were much lower than would be expected from the annexin V assay, with no significant difference between the β-thalassaemia and control groups, the increased PS-bearing erythroblasts observed in murine β-thalassaemic bone marrow might result from mechanisms other than apoptosis.

### Increased autophagy in murine β-thalassaemic erythroblasts

**Figure 2:**
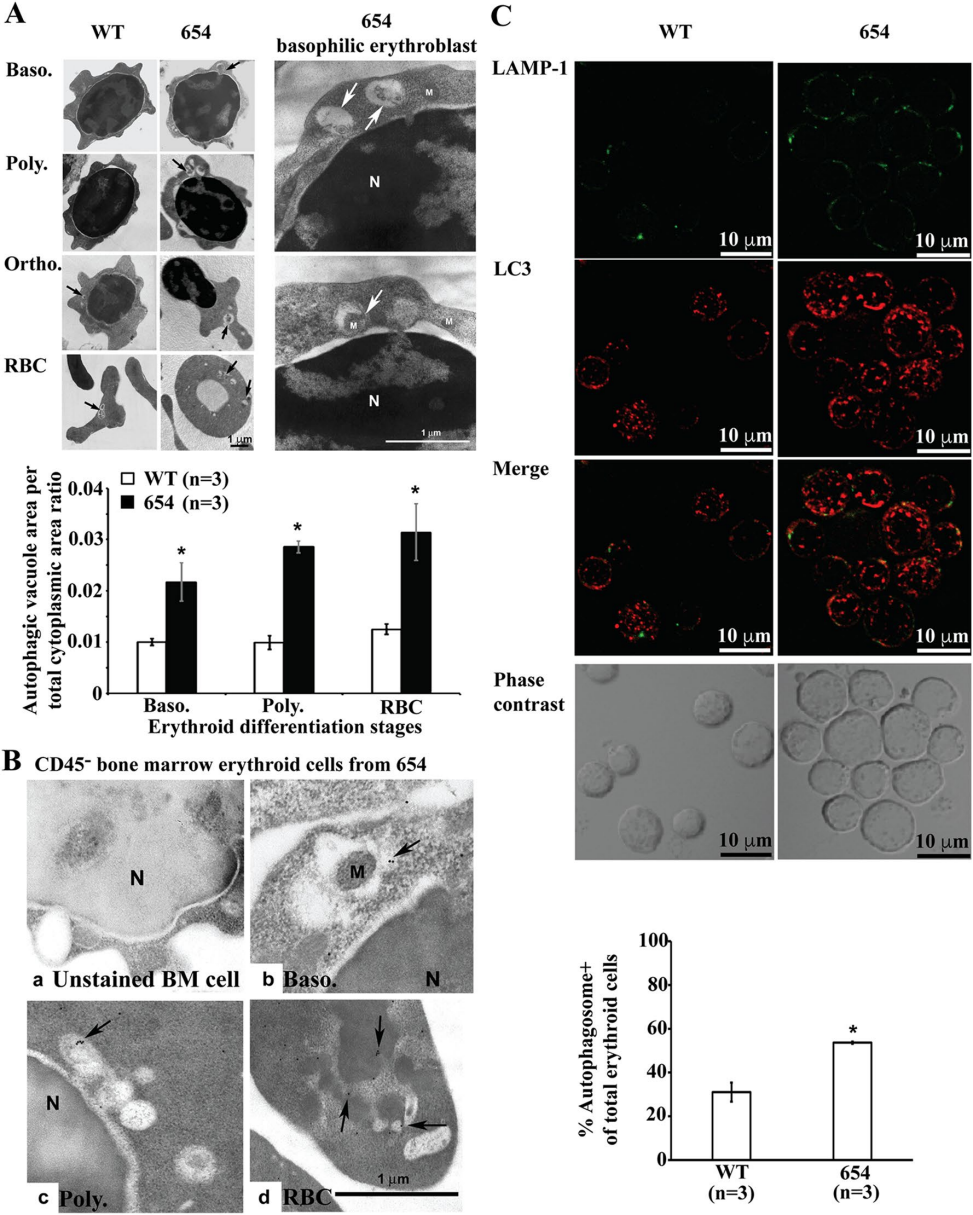
Elevated autophagy in β-thalassaemic erythroblasts. (**A**) Increased autophagic vacuoles in murine β-thalassaemic erythroblasts. Ultrastructural analysis of murine CD45^–^ bone marrow erythroid cells from β-thalassaemic mice (654, middle column) and wild type mice (WT, left column) were evaluated for autophagic vacuoles (arrow) in erythroid subpopulations using transmission electron microscope. High magnification of electron micrographs from basophilic erythroblasts of β-thalassaemic mice is shown in the right-column. The illustration was captured and analysed for the area of the individual autophagic vacuole and total cytoplasmic area using Scion Image software. Quantitative analysis of autophagic vacuole area per total cytoplasmic area ratio is presented as mean ± SEM. *Significantly different when compared to wild type mice at *P* < 0.05, using T-test. N, nucleus; M, mitochondria; Baso, basophilic erythroblasts; Poly, polychromatophilic erythroblasts; Ortho, orthochromatic erythroblasts and RBC; red blood cells. (**B**) Localization of LC3 in autophagosomes as determined by immunogold electron microscope. CD45^–^ bone marrow erythroid cells from β-thalassaemic mice were stained with LC3 using an immunogold assay to determine LC3 (arrow) in the membrane of autophagosomes in (a) unstained CD45^–^ bone marrow cells, (b) basophilic erythroblasts, (c) polychromatophilic erythroblasts and (d) RBC. N; nucleus, M; mitochondria. (**C**) Increased autophagy in murine β-thalassaemic erythroblasts was determined by confocal microscopy. Analysis of LAMP-1 (green) and LC3 (red) in CD45^–^ bone marrow erythroid cells from β-thalassaemic mice (654, right-column) and wild type mice (WT, left-column) was undertaken using a confocal microscope. Number of autophagosomes positive erythroblasts was determined by LC3 puncta and calculated as percentages of autophagic cells in total erythroid cells (Supplementary Fig. S4). Data are presented as mean ± S.D. *Significantly different when compared to wild type mice at *P* < 0.05, using T-test.

The presence of autophagic vacuoles, membrane-bound vacuole containing intracellular contents material, were examined in murine CD45^–^ bone marrow erythroblasts by transmission electron microscopy. An elevated autophagic vacuole area per total cytoplasmic area in β-thalassaemic mice erythroblasts was observed from the basophilic erythroblast stage to the stage of terminal differentiation (Fig. 2A). However, the number of autophagic vacuoles per total cytoplasmic area was not significantly different between β-thalassaemic mice and wild type mice. During autophagosome formation, the full length free cytosolic form of LC3-I, is cleaved and modified by conjugation with phosphatidylethanolamine (PE) to generate the lipid-conjugated autophagosome-associated form, LC3-II^19^. The presence of autophagosomes in β-thalassaemic erythroblasts was confirmed by the presence of LC3 on the autophagosomes using immunogold electron microscopy (Fig. 2B). An increased number of autophagosome positive erythroblasts in β-thalassaemic erythroblasts was supported by increased LC3 puncta as determined by confocal microscopy (Fig. 2C). The co-localization of LC3 and LAMP-1 (a lysosomal marker) in CD45^–^ bone marrow erythroblasts was observed, indicating fusion between autophagosomes and lysosomes to form autolysosomes. These results indicate increased autophagosome numbers in murine β-thalassaemic erythroblasts.

**Figure 3:**
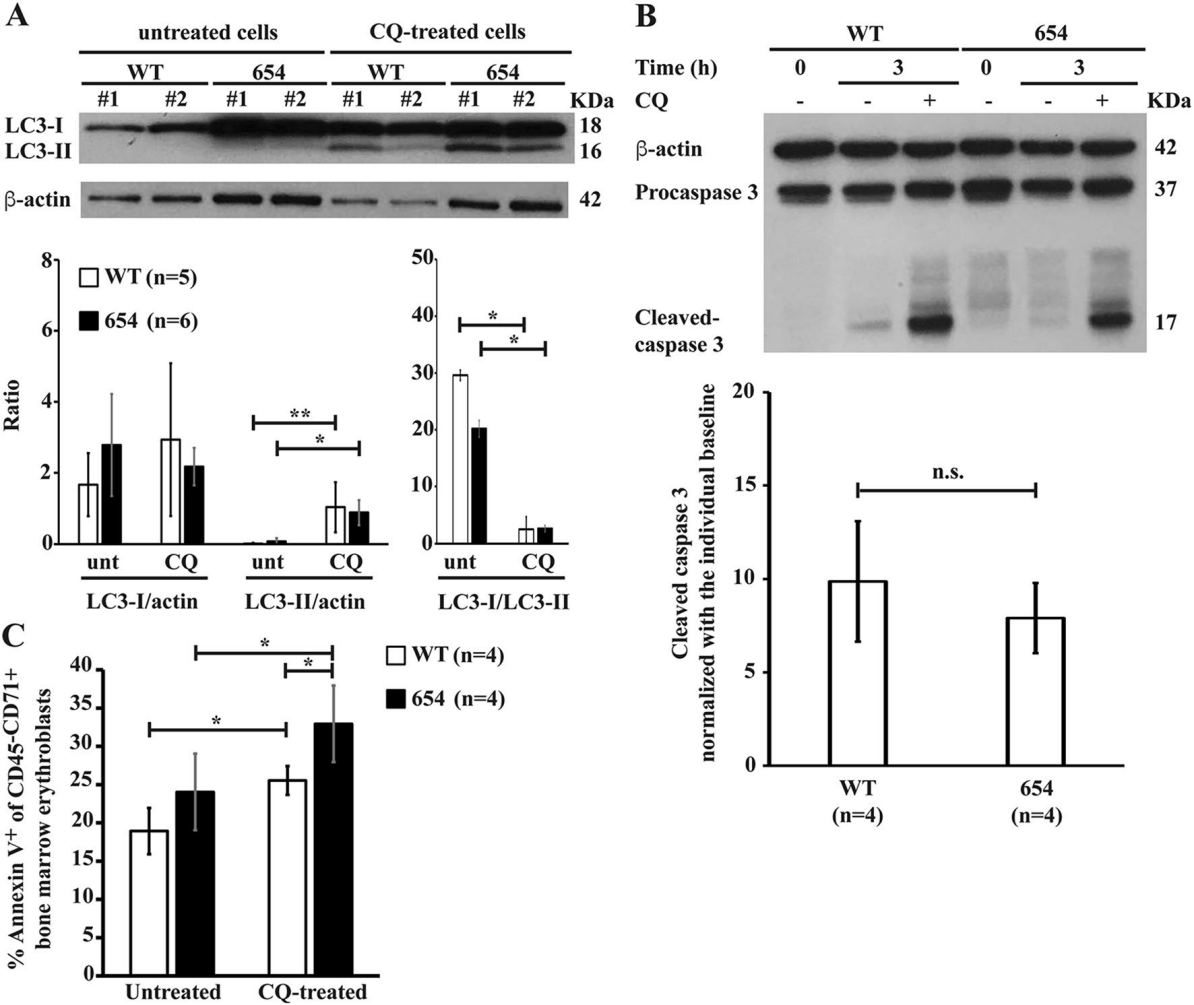
Inhibition of autophagy accelerates apoptotic cell death. CD45^–^CD71^+^bone marrow erythroblasts from β-thalassaemic mice (654) and wild type mice (WT) were incubated with or without the presence of 100 μM chloroquine (CQ) to inhibit autophagic flux at 37 °C, 5% CO_2_. Untreated CD45^–^CD71^+^ bone marrow erythroblasts were cultured in IMDM media supplemented with 20%FBS at 37 °C, 5% CO_2_ for 3 and 24 h as a control. (**A**) Increased LC3-II levels after inhibiting autophagic flux with chloroquine. LC3-I and LC3-II expression in untreated and chloroquine-treated CD45^–^CD71^+^ bone marrow erythroblasts at 3 h. after treatment were determined using western blot analysis (Supplementary Fig. S5). LC3-I and LC3-II expression were normalised with β-actin, then, the ratio of LC3-I and LC3-II was calculated. (**B**) Inhibition of autophagic flux leads to increased cleavage of caspase 3. Cleaved caspase 3 and procaspase 3 at 3 h. after chloroquine treatment of CD45^–^CD71^+^ bone marrow erythroblasts were determined by western blot analysis (Supplementary Fig. S6). Cleaved caspase 3 expression was normalised with β-actin, then the ratio of cleaved caspase 3 in chloroquine-treated erythroblasts was normalised with the individual untreated erythroblasts. (**C**) Increased annexin V^+^ erythroblasts after autophagic flux inhibition. The percentages of PS-bearing CD45^–^CD71^+^ bone marrow erythroblasts were determined at 24 h after chloroquine treatment using fluorochrome conjugated annexin V and flow cytometry. Data are presented as mean ± S.D. *Significantly different when compared between groups at *P* < 0.01, using T-test. **Significantly different when compared between groups at *P* < 0.05, using T-test.

Observation of increased autophagosomes is not an indicator of increased autophagy activity. The accumulation of autophagosomes as indicated by co-localization of LC3 and LAMP-1 in β-thalassaemic mice erythroblasts may be caused by the increased formation of autophagosomes and/or blockage of autophagosomal maturation and/or inhibition of autophagic flux. Thus, autophagic flux was determined by using chloroquine, which inhibits the autolysosome contents degradation via increasing lysomal pH and inhibiting acidic lysosomal proteases. During autophagosome formation, LC3-I is conjugated to PE to form LC3-II and recruited to autophagosomal membranes. LC3-II and other intra-autophagosomal components are digested after fusion of the autophagosomes with lysosomes to form autolysosomes. CD45^–^CD71^+^ bone marrow erythroid cells were treated with chloroquine for 3 h. Western blot analysis showed nearly undetectable LC3-II in untreated erythroblast of both groups (Fig. 3A). Significantly increased levels of LC3-II in chloroquine treated bone marrow erythroblasts were observed in both β-thalassaemic and wild type mice. In addition, decreased LC3-I/LC3-II was observed in chloroquine treated erythroblasts, confirming the presence of autophagic flux and that the low level of LC3-II was due to lysosome-dependent degradation. The effect of autophagic flux inhibition on apoptosis was then examined. Chloroquine treatment caused significantly increased caspase 3 activation in erythroblasts from both β-thalassaemic and wild type mice compared to untreated erythroblasts (*P* < 0.05) but no significant differences were observed in caspase 3 activation between β-thalassaemic and wild type erythroblasts treated with chloroquine (Fig. 3B). In addition, after 24 h treatment with chloroquine, PS-bearing erythroblasts were also increased with the effect being more pronounced in β-thalassaemic erythroblasts (Fig. 3C)**,** suggesting that autophagy promotes erythroblasts survival.

### Increased autophagy in bone marrow erythroblasts of β-thalassaemia/HbE patients

**Figure 4:**
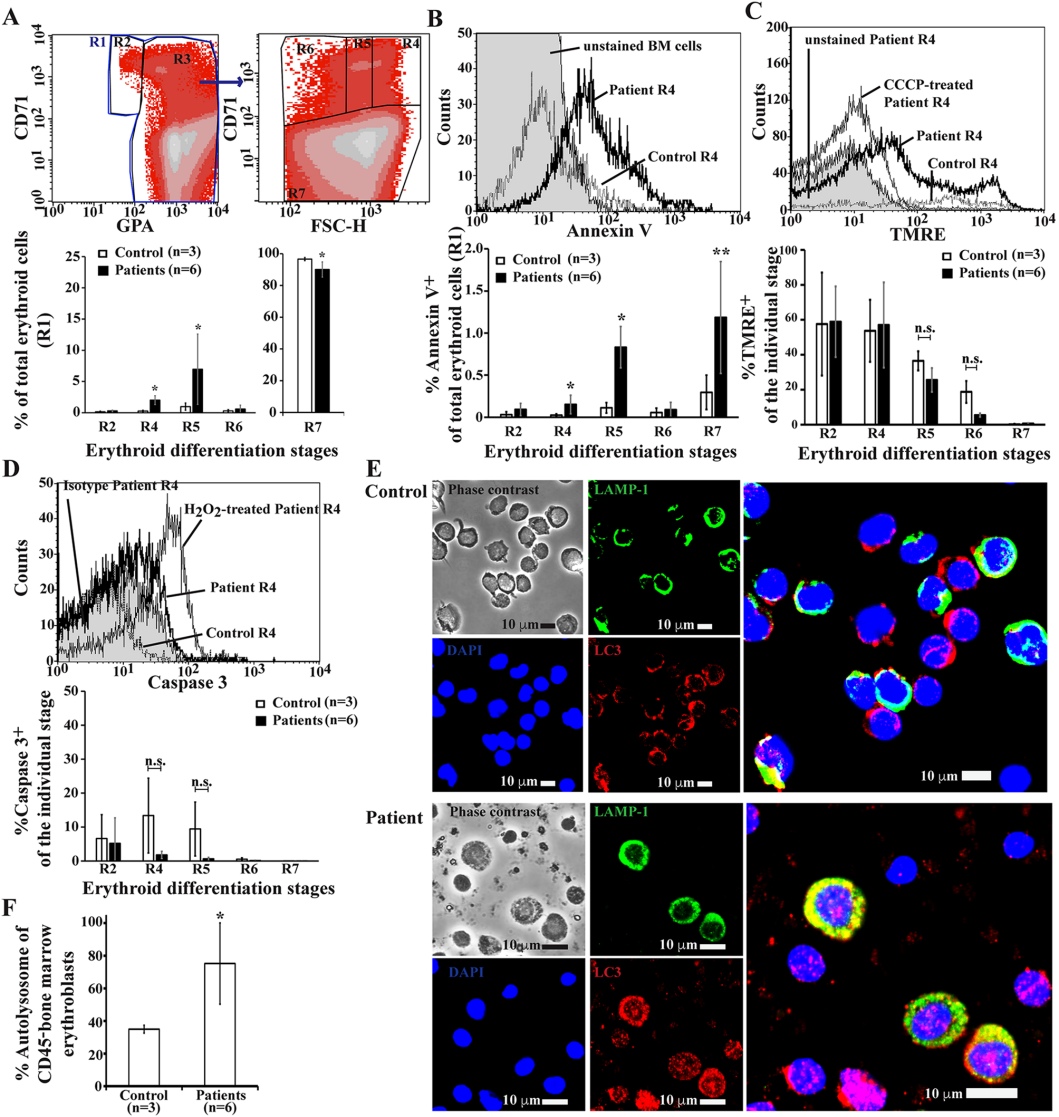
Elevated PS-bearing erythroblasts and autophagy without increased apoptosis in bone marrow of β-thalassaemia/HbE patients. (**A**–**D**) Apoptotic markers were examined in whole bone marrow erythroid cells from β-thalassaemia/HbE patients using flow cytometry. (**A**) Definition of flow cytometric erythroblast subsets. Bone marrow cells were labeled with monoclonal antibodies specific to glycophorin A (GPA, an erythroid cell marker) and CD71 and further analysed with respect to their forward scatter (FSC-H) (Supplementary Table 1). GPA^+^ erythroid cells in the R1 region were divided into two subpopulations as GPA^dim^CD71^+^ erythroblasts in the R2 region (proerythroblasts as the majority cell type) and GPA^high^ erythroid cells (R3 region). Then, the R3 region was divided into a R4 region (basophilic erythroblasts as the majority cell type), a R5 region (polychromatophilic erythroblasts as the majority cell type), a R6 region (orthochromic erythroblasts and reticulocytes as the majority cell types) and a R7 region (red blood cells as the majority cell type) using CD71/FSC-H. The percentages of erythroid differentiation were analysed using a BD FACSCalibur flow cytometer and CellQuest Pro™ software. Percentages of (**B**) PS exposure (annexin V^+^ cells), (**C**) mitochondrial transmembrane potential (TMRE^+^ cells) were determined, and (**D**) activated caspase 3, as assessed by intracellular staining using a PE conjugated anti-human active caspase 3 antibody, in each erythroblast subpopulation was determined (Supplementary Fig. S7). Unstained human bone marrow cells were used as a negative control to determine annexin V and TMRE signal. Human bone marrow cells were treated with 100 μM carbonyl cyanide 3-chlorophenylhydrazone (CCCP) for 30 min at 37 °C, 5% CO_2_ to directly disrupt mitochondrial transmembrane potential. Human bone marrow cells treated with 193.8 mM hydrogen peroxide (H_2_O_2_) for 15 min at 37 °C, 5% CO_2_ were used as a positive control for activated caspase 3. Human bone marrow cells stained with an isotype control were used as a negative control for determining activated caspase 3. (**E**) Co-localization of LC3 (red) and LAMP-1 (green) in CD45^–^ human bone marrow erythroblasts was analyzed. DAPI (blue) was used as nucleus marker. The purity of human CD45^–^ bone marrow erythroblasts was 45–70% as measured by flow cytometry. The LAMP1 unstained cells were lymphocytes, which were excluded from the analysis (Supplementary Fig. S8). (**F**) The percentages of autophagic cells as Pearson’s correlation coefficient between LC3 and LAMP-1 at cut-off ≥ 0.5 were calculated (Supplementary Fig. S9). The data are presented as mean ± S.D. *Significantly different when compared to wild type mice at *P* < 0.01, using Mann–Whitney U test. **Significantly different when compared to wild type mice at *P* < 0.05, using Mann–Whitney U test.

To evaluate whether our findings in β-thalassaemic mice are reflected in β-thalassaemia/HbE patients, PS exposure, mitochondrial transmembrane potential, active caspase 3 and co-localization of LC3 and LAMP-1 in human CD45^–^ bone marrow erythroid cells were determined. Similar to the results seen in β-thalassaemic mice, increased amounts of basophilic erythroblasts and polychromatophilic erythroblasts, but decreased amount of the terminal differentiated stage of erythroid cells in β-thalassaemia/HbE patients, an indication of ineffective erythropoiesis, were observed when compared to control subjects (Fig. 4A). The R7 region was spread from low to high FSC-H, similar to the R4 region, suggesting marked anisocytosis. Increased PS-bearing cells in R4 and R5 regions with a majority of basophilic erythroblasts and polychromatophilic erythroblasts in β-thalassaemia/HbE patients as compared to cells from control subjects was also observed (Fig. 4B). Notably, mitochondrial transmembrane potential was not significantly different between groups (Fig. 4C). The percentages of TMRE^+^erythroid cells in R5 and R6 with a majority of polychromatophilic erythroblasts and orthochromic erythroblasts in β-thalassaemia/HbE patients seemed to be reduced when compared to control group, although the absence of statistically significant differences could be related to the small number of subjects in the study. To determine the extent of apoptosis in β-thalassaemia/HbE erythroblasts, activation of caspase 3 as an effector caspase was examined and the results showed minimal caspase 3 activation with no significant difference to that of controls (Fig. 4D and Supplementary Fig. S7). Autophagy in CD45^−^ bone marrow erythroblasts from β-thalassaemia/HbE patients was then investigated. LAMP-1 was uniformly distributed in the cytosol. Therefore, co-localization of LC3 and LAMP-1 was quantified by determining the Pearson’s correlation coefficients of co-localization between LC3 and LAMP-1, with a cut-off at ≥ 0.5 (Supplementary Figs. S8 and S9). Interestingly, co-localization in human bone marrow erythroblasts from β-thalassaemia/HbE patients was significantly increased as compared to cells from control subjects (Fig. 4E, F).

## Discussion

Ineffective erythropoiesis in β-thalassaemia is a key factor that results from erythroblast death in the bone marrow, leading to severe anaemia and other complications. It is characterized by accelerated erythroid proliferation, expanded erythroid progenitors, maturation blockage at the stage of basophilic and polychromatophilic erythroblasts, resulting in only a limited number of mature red blood cells being produced. The pathological mechanisms of dyserythropoiesis are complex and are still not fully understood. Increased apoptosis of erythroid precursors combined with decreased differentiation has been proposed to be responsible for the ineffective erythropoiesis in β-thalassaemia^2–6^. However, the percentage of cells undergoing apoptosis^2–5^ is relatively low compared with the extreme expansion of medullary and extramedullary erythroid progenitors, and as predicted by ferrokinetic studies^2^. Herein, we report an increase in autophagy in erythroid progenitor cells in bone marrow of both β-thalassaemic mice and β-thalassaemia/HbE patients.

The β^IVS2-654^-thalassaemic mouse model has been developed by gene targeting to replace the two murine adult β-globin genes, β^maj^ and β^min^, with a copy of the human β^IVS2-654^-thalassaemic gene. Heterozygous β^IVS2-654^-thalassaemic mice recapitulate the characteristics of β-thalassaemia patients including anaemia, abnormal red blood cell indices, increased ROS levels in red blood cells, increased red blood cell-derived vesicles, splenomegaly, iron overload and osteoporosis^20–22^. The mouse model was also used as model for the study of a novel therapy for β-thalassaemia through restoration of β-globin pre-mRNA splicing using splice switching oligonucleotide^23,24^.

Although the spleen is the main erythropoiesis site in compensation for anaemia in mice including thalassaemic mice, in this study, the bone marrow was used as representative of erythropoiesis in thalassaemic mice. As the fundamental cause of erythroblast abnormality in β-thalassaemia is imbalance globin chain synthesis, an intrinsic factor, this should not cause major differences between erythroblasts in the bone marrow and the spleen of β-thalassaemic mice. Both bone marrow and spleen of β-thalassaemic mice have similar erythroid hyperplasia at the basophilic and polychromatophilic erythroblast stages and decreased orthochromic erythroblasts, with significantly increased PS exposure at the basophilic erythroblast stage^21^. Comparable expression of genes such as erythroid regulators of hepcidin (*Erfe, Gdf15* and *Twsg1*) between bone marrow and spleen of several mouse models of anaemia including thalassaemic mice have been reported^25^. Moreover, the cell-surface death receptor, Fas, and its ligand, FasL, at each erythroblast stage were similar between the bone marrow and spleen of thalassaemic mice^26^.

Flow cytometric analysis of erythroid subpopulation here was performed by determining expression level of the transferrin receptor (CD71) and an erythroid lineage specific marker (TER119 for murine or GPA for human) coupled with determination of size using FSC-H. Although, CD71 is widely used for distinguishing between different stages of erythroid differentiation, the method is less effective in discriminating between erythroblast stages, with each gated population showing mixed erythroid subpopulations especially for late stage erythroblasts^18^. Better discrimination between different stages of erythroid differentiation by flow cytometry have been reported using CD44/TER119/FSC-H and integrin-IV/Band 3 for murine and human, respectively^18,27^. The use of CD71/TER119/FSC-H might be the cause of the high variability seen for mitochondrial potential and caspase 8 and 9 activation. Another limitation of the flow cytometric analysis used in this study was that dead cells were not excluded. A Combination of annexin V with propidium iodide or 7ADD that can distinguish between early and late apoptotic cells was not used in this study. Additionally, the analysis of human erythroblast activated caspase 3 used cytoFix/Perm solution for the intracellular staining to allow the entrance of the anti-active caspase 3 monoclonal antibody which may have affected the morphological parameters both of FSC and SSC of the cells, resulting in difficulty in the recognition of the specific subpopulations by flow cytometry.

Increased PS-bearing basophilic erythroblasts in the bone marrow from both β-thalassaemia mice and β-thalassaemia/HbE patients was observed, consistent with previous reports^2,4,6,21^. Remarkably, minimal apoptotic activation of both the extrinsic death receptor pathway and the intrinsic mitochondrial pathway was observed in bone marrow erythroblasts from β-thalassaemic mice and β-thalassaemia/HbE patients. A previous study on the bone marrow of β-thalassaemia patients also found limited evidence of activation of the mitochondrial apoptosis pathway as determined by mitochondrial potential, and only slightly increased caspase 9 activation^28^. However, that study observed increased Fas and FasL expression and elevated levels of both annexin V and Fas or FasL in erythroblasts from β-thalassaemia patients. In contrast, murine β-thalassaemic bone marrow and spleen showed both Fas and FasL were downregulated in proerythroblasts and basophilic cells^26^.

Caspase 3 is required for normal development and enucleation. Inhibition of caspase 3 in human erythroid cells leads to a blockage of cell differentiation from proerythroblasts to basophilic erythroblasts^29^. Caspase 3 mediating nuclear opening with the consequent release of histones into the cytoplasm is a critical step of chromatin condensation during murine erythropoiesis^30^. The caspase 3 activation is transient and highly different in the individual erythroblast stages. A previous study showed levels of active cleaved caspase 3 peaked in early erythropoiesis at the erythroid colony forming unit (CFU-E) stage, after which protein levels dropped below the limit of detection by western blot analysis^29^. In this study, the lack of detection of cleaved murine caspase 3 might be due to the limiting sensitivity of western blot analysis. In addition, the analysis was undertaken on total CD45^−^CD71^+^ bone marrow erythroblasts, a mix of erythroblast stages with varying levels of caspase 3 activation. Notably, a low level of activated caspase 3 was detected in β-thalassaemia/HbE erythroblasts by flow cytometry using a monoclonal antibody specific to activated caspase 3, but with no significant difference from controls.

Autophagy plays an important role in maintaining homeostasis in mammalian cells through recycling of cytoplasmic constituents via formation of autophagosomes that sequester cell organelles and cytoplasmic materials and subsequently fuse with lysosomes to degrade the intra-autophagosome components. During terminal erythroid differentiation, erythroblasts requires autophagy to eliminate mitochondria and ribosomes after enucleation^15,16^. Our previous study showed a significantly higher level of autophagy in cultured erythroblasts derived from β-thalassaemia/HbE patient peripheral blood CD34^+^ cells as compared to the same cells derived from normal controls^17^. In this study, increased autophagy as demonstrated by increased autophagic markers such as autophagosome area, LC3 expression on autophagosomes, increased LC3-II and co-localization of LC3 and LAMP-1 in the cytoplasm was observed in bone marrow cells of β-thalassaemic mice and β-thalassaemia/HbE patients as compared to control cells. Although autophagy is essential for the maintenance of cellular homeostasis, excessive stress could be able to induce autophagy-dependent cell death. Autosis is an autophagy-dependent, non-apoptotic and non-necrotic form of cell death^31^. Autotic cells display a dramatic increase in the number of autophagosomes and autolysosomes, electron dense mitochondria, fragmented and swollen ER, nuclear membrane convolution and focal swelling of the perinuclear space^31^. However, we did not observe any of the characteristics of autotic cell death in β-thalassaemic mice erythroblasts observed by transmission electron microscopy.

Under pathophysiological conditions, β-thalassaemic erythroblasts with defective β-globin chain synthesis, generate an excess of unbound α-globin chains. Accumulation and precipitation of excess α-globin chains and heme released from denatured excess α-globin chains could generate cellular stress and trigger apoptosis. However, there is no evidence to indicate a massive elevation of apoptosis in β-thalassaemic erythroid cells. Degradation of unbound α-globin via the UNC1-like kinase 1 (ULK1)-dependent autophagy has been showed in β-thalassaemic mice erythroblasts^32^. Inhibition of the autophagy inhibitor mTORC1 by rapamycin reduced the accumulation of insoluble unbound α-globin chains^32^. In addition, inhibition of lysosomal acidification with chloroquine inhibits α-globin chain degradation^33^. Interestingly, thalassaemic mice treated with rapamycin showed reduced red blood cell pathology^32^. However, apoptosis was not investigated in these studies. Here, inhibition of autophagy by chloroquine lead to increased apoptosis. Thus, we hypothesized that autophagy is necessary for processing unbound α-globin and preventing increased apoptosis in β-thalassaemic erythroblasts, and inhibition of autophagy would lead to accumulation of α-globin and enhanced apoptosis.

The mechanism responsible for PS exposure in β-thalassaemic erythroblasts is not thoroughly understood. The regulation of membrane lipid asymmetry is controlled by specific lipid transporters, and lowered amino-phospholipid translocase activity and increased Ca^2+^ dependent phospholipid scramblase activity can lead to PS exposure^34^. Increased scramblase activity in murine β-thalassaemia erythroid cells has been previously demonstrated^35^. Additionally, a majority of β-thalassaemic erythroid cells with PS exposure have activated phospholipid scramblase. Our previous study demonstrated that levels of Ca^2+^ were significantly elevated in β-thalassaemia/HbE erythroblasts^17,36,37^. Interestingly, reduction of Ca^2+^ in β-thalassaemia/HbE erythroblasts by treatment with ethylene glycol tetraacetic acid (EGTA) reduced the level of PS exposure to approximately normal control levels^17^. In erythroblastic islands, PS is a signal for engulfment and phagocytosis by macrophages of nuclei expelled from erythroblasts^38^. PS exposure on β-thalassaemic erythroblasts in bone marrow could also mark these cells as a phagocytotic targets for rapid clearance by macrophages. Moreover, macrophages in β-thalassaemia/HbE patients are also strongly activated^39^. Notably, enhanced phagocytosis of β-thalassaemic erythroblasts has been reported, and this is mediated by the exposure of PS on the β-thalassaemic erythroblasts as PS carrying vesicles and annexin V strongly inhibited phagocytosis^40^.

Ineffective erythropoiesis is key factor leading to severe anaemia and other complications in β-thalassaemia. The mechanism of ineffective erythropoiesis is still not clearly documented. Our data provides some evidence that autophagy could have a major role in ineffective erythropoiesis in β-thalassaemia, with elevated autophagy, an intrinsic safety mechanism, in response to the cellular stress, leading to minimal apoptosis. However, increased PS exposure occurring via other mechanisms might be the cause of their rapid phagocytic removal by macrophages, and consequently ineffective erythropoiesis in β-thalassaemia.

## Methods

### Animals

This study was approved by the Mahidol University Animal Care and Use Committee (approval number MU-ACUC 2009/001). All methods were performed in accordance with the relevant guidelines and regulations. Wild type mice and β^IVS2-654^-thalassaemia in C57Bl/6 background mice^20^ were employed in the present study. Murine bone marrow cells were harvested from femurs and tibias as described in a previous study^21^. All methods are reported in accordance with Animal Research: Reporting of In Vivo Experiments (ARRIVE) guidelines and American Veterinary Medical Association (AVMA) Guidelines for the Euthanasia of Animals (2020).

### Flow cytometric analysis of murine erythroid apoptosis

Erythroid subpopulations were defined as described in previous studies^18,26^. Whole bone marrow samples were stained with fluorochrome conjugated monoclonal antibodies specific to CD71 (transferrin receptor, BD Biosciences, San Jose, CA) and TER119 (red cell marker, BD Biosciences). Then, samples were combined with a third-color fluorescent channel staining such as fluorochrome conjugated annexin V (phosphatidylserine marker, BD Biosciences) or 100 nM 3,3′-dihexyloxacarbocyanine iodide (DiOC_6_(3)) (Sigma-Aldrich, St. Louis, MO) to determine mitochondrial transmembrane potential. For determination of activated caspase 8 and caspase 9 in living cells, bone marrow samples (2 × 10^6^ cells) were incubated with either fluorescein isothiocyanate (FITC)-conjugated IETD-FMK (for caspase 8, Calbiochem, San Diego, CA) or sulforhodamine (Red)-conjugated LEHD-FMK (for caspase 9, Calbiochem) following the manufacturer’s recommendation. The cocktail of monoclonal antibodies that was used in this study are shown in Supplementary Table 1. Data of 100,000 events was acquired and analysed using a BD FACSCalibur flow cytometer and CellQuest Pro™ software (BD Biosciences). K562 erythroleukemic cells treated with 193.8 mM hydrogen peroxide (H_2_O_2_) for 15 min at 37 °C, 5% CO_2_ were used as a control.

### Murine bone marrow erythroblast isolation

CD45^−^ erythroblasts and CD45^–^CD71^+^ erythroblasts from bone marrow samples were isolated using a MACS cell separation system (Miltenyi Biotec, Bergen Gladbach, Germany) as the manufacturer’s instructions. The purity of murine CD45^–^CD71^+^ bone marrow erythroblasts was 73–91% as measured by flow cytometry.

### Transmission electron microscopic analysis of murine erythroid autophagy

Bone marrow erythroblasts were harvested and analysed for morphology and LC3 expression using a transmission electron microscope^41^. CD45^–^ bone marrow erythroid cells (1 × 10^7^ cells) were fixed in 2.5% glutaraldehyde solution for 4 h at 4 °C, then, washed with Sorenson’s phosphate buffer and stained with 1% Caulfield’s osmium tetroxide supplemented with sucrose for 1 h. Samples were dehydrated with different concentrations of ethanol and finally propylene oxide, then, embedded in Embed 812 resin BEEM capsules (Electron Microscopy Science, Hatfield, PA) at 65–80 °C for overnight. Thin section (60–90 nm) was collected and mounted on copper grids, stained with uranyl acetate, and counterstained with lead citrate. Grids were observed and images were obtained using a transmission electron microscope (Hitachi H-7100, Hitachi High-Tech Science Corporation, Tokyo, Japan) operated at 100 kV. Images were captured at 4000 × magnification for 10 fields per sample by sequential field collection. Autophagic vacuoles/autolysosomes was defined as membrane-bound vacuole containing electron-dense cytoplasmic material and/or organelles at various stages of degradation. The electron-lucent cleft between the two limiting membranes was used to aid identification. The area of autophagic vacuoles and the total cytoplasmic area of erythroblasts were measured using the Scion Image Beta 4.02 analysis software (Scion Corporation, Chicago, IL). A total of 50 cells per sample were analysed.

For LC3 expression determination by immunogold staining^41^, a thin section of CD45^–^ bone marrow erythroid cells from β-thalassaemic mice processed for transmission electron microscopy were collected and mounted on nickel grids. Grids were blocked with 0.5% BSA-C (Aurion, Wageningen, Netherlands), and then stained with a rabbit anti-LC3 polyclonal antibody (Aurion) as a primary antibody and gold-conjugated F(ab)_2_ fragments of goat anti-rabbit IgG (Aurion) as a secondary antibody, and counterstained with uranyl acetate. Control samples used rabbit serum (Dako, Glostrup, Denmark) in place of the primary antibody were used as a negative control. Images were captured using a transmission electron microscope (Hitachi H-7100) operated at 100 kV.

### Confocal microscopic analysis of murine erythroid autophagy

CD45^–^ bone marrow erythroid cells (3 × 10^5^ cells) were fixed with 4% paraformaldehyde and blocked with 1% BSA-C solution (Aurion) for 1 h. Subsequently, samples were permeabilized with 0.3% Triton X-100 in phosphate buffered saline and incubated with a rabbit anti-LC3 polyclonal antibody (Novus Biologicals, Littleton, CO), then, subsequently a Cy5-conjugated goat anti-rabbit IgG (H+L) F(ab’)_2_ fragments (Invitrogen, Carlsbad, CA). After incubation and wash, samples were stained with FITC conjugated rat anti-LAMP-1 monoclonal antibody (Biolegend, San Diego, CA). Fluorescent signal was observed using an Olympus FluoView 1000 confocal microscope (Olympus, Tokyo, Japan). Illustration was captured using a 60 × objective lens for 10 field per sample by sequential field collection and discard 10 fields. A total of 50 cells per sample were analysed. Image analysis and calculation of Pearson's correlation coefficients and confidence intervals were undertaken as previously described^17,42^. Autophagosomes with punctate LC3 expression in erythroblasts were count and calculated as the percentage of autophagic cells in total erythroid cells (Supplementary Fig. S4).

### Inhibition autophagic flux of murine erythroblasts

CD45^–^CD71^+^ bone marrow erythroblasts were cultured in Iscove’s Modified Dulbecco’s medium (IMDM) supplemented with 20% fetal bovine serum (FBS) with or without 100 μM chloroquine at 37 °C, 5% CO_2_. Chloroquine was dissolved in culture medium and filtered through a 0.2 μM filter before use. The cells were collected at 3 h. after treatment for analysis of LC3-I and LC3-II by western blot^43^ and at 24 h. for determination of PS expose by flow cytometry.

### Western blot analysis of erythroid autophagy

CD45^–^CD71^+^ bone marrow erythroid cells were lysed and sonicated in lysis buffer (50 mM Tris (pH 7.4), 150 mM NaCl, 0.1% SDS and 1% Triton X-100) supplemented with protease inhibitor cocktail (Sigma-Aldrich). A total of 50 μg protein of the lysates was separated through 15% denaturing polyacrylamide gels and transferred to polyvinylidene difluoride membrane (Bio-Rad Laboratories). Primary antibodies used were a rabbit anti-caspase 3 polyclonal antibody (Cell Signaling Technology, Danvers, MA), a rabbit anti-LC3 polyclonal antibody (Abcam, Cambridge, UK) and a rabbit anti-β-actin monoclonal antibody (Sigma-Aldrich). Secondary antibody was used was a horseradish peroxidase conjugated goat anti-rabbit IgG (Sigma-Aldrich). The densitometric analysis was performed using ImageJ software (The National Institutes of Health, MD).

### Human bone marrow collection

This study was performed in accordance with the Helsinki declaration and was approved by the Mahidol University Institutional Review Board (approval number MU-CIRB 2014/031.1703). Written informed consent was obtained from all individual participants in this study. Patients under treatment with hydroxyurea, aspirin, antibiotics, anti-depressants, beta-blockers and anti-platelets were excluded, and no participant had been hospitalised or transfused within 4 weeks of sample collection. Human bone marrow samples were collected from 6 β-thalassaemia/HbE patients and 3 control subjects into citrate phosphate dextrose adenine solution anticoagulant by using the bone marrow aspiration technique. Samples were processed within 1 h after aspiration. All methods were performed in accordance with the relevant guidelines and regulations. Haematological parameters are shown in Supplementary Table 2.

### Human bone marrow erythroblast isolation

CD45^–^ erythroblasts from bone marrow samples were isolated using a MACS cell separation system (Miltenyi Biotec) as the manufacturer’s instructions. The purity of human CD45^–^ bone marrow erythroblasts was 45–70% as measured by flow cytometry.

### Flow cytometric analysis of human erythroid apoptosis

Erythroid subpopulations were defined as described in previous studies^17,27^. Whole bone marrow samples were stained with fluorochrome conjugated monoclonal antibodies specific to CD45 (leukocyte marker, BD Biosciences), CD71 (BD Biosciences) and glycophorin A (GPA, red cell marker, BD Biosciences). Then, samples were combined with a fourth-color fluorescent channel staining such as fluorochrome conjugated annexin V (BD Biosciences) or 50 nM tetramethylrhodamine ethyl ester perchlorate (TMRE) (Sigma-Aldrich) for mitochondrial transmembrane potential. Whole bone marrow samples were incubated with 100 μM carbonyl cyanide 3-chlorophenylhydrazone (CCCP) for 30 min at 37 °C, 5% CO_2_ for direct disruption of mitochondrial transmembrane potential and this was used as a control.

For intracellular staining to determine active caspase 3, whole bone marrow samples were incubated with cold-cytoFix/Perm (BD Biosciences) for 10 min at 4 °C and then were stained with fluorochrome conjugated monoclonal antibodies specific to CD45, CD71, GPA and either activated caspase 3 (BD Biosciences) or isotype control for 30 min at 4 °C. Data of 100,000 events was acquired and analysed using a BD FACSCalibur flow cytometer and CellQuest Pro™ software. Bone marrow sample treated with 193.8 mM H_2_O_2_ for 15 min at 37 °C, 5% CO_2_ was used as positive control. The cocktail of monoclonal antibodies that was used in this study are shown in Supplementary Table 1.

### Human erythroid cell autophagic analysis

CD45^–^ human bone marrow erythroblasts were isolated and analysed co-localization of LC3/LAMP-1 using confocal microscope^17,42^. CD45^–^ human bone marrow erythroblasts (3 × 10^5t5^ cells) were fixed and stained with a rabbit anti-LC3 polyclonal antibody (Novus Biologicals), then, subsequently a Cy5-conjugated goat anti-rabbit IgG (H+L) F(ab’)_2_ fragments (Invitrogen) as secondary antibody, after incubation and wash, samples were incubated with a FITC conjugated rat anti-LAMP-1 monoclonal antibody (Biolegend). After incubation and wash, samples were stained with 4′,6-Diamidino-2-phenylindole, dihydrochloride (DAPI, Molecular Probes, Eugene, OR). Images were captured using a 60 × objective lens for ≥ 10 field per sample. A total of 50 erythroid cells per sample were analysed. Fluorescent signal was observed and analysed as described in the section of “Confocal microscopic analysis of murine erythroid autophagy” (Supplementary Figs. S8 and S9).

### Statistical analysis

Data were analyzed using SPSS Version 18.0 (SPSS Inc., Chicago, IL). Comparisons between parameters which analysed using flow cytometry and cell counting were evaluated by Mann–Whitney U test. Comparisons between parameters which image analysis including autophagic vacuole per cytoplasmic area ratio and densitometric analysis of protein expression using Western blot were evaluated by independent Student’s t-tests. The threshold for statistical significance for all comparisons was *P* < 0.05.

## Supplementary Information

Supplementary Information.

## Data Availability

All data related to this study can be obtained on request, while all the analysed data were included in this published article and its supplementary information files.

## Abbreviation

LC3 or MAP1LC3B: Microtubule-associated protein 1A/1B light chain 3B
