# Supplementary material for: Increased autophagy leads to decreased apoptosis during β-thalassaemic mouse and patient erythropoiesis
Source: Sci Rep. 2022 Nov 3;12:18628. doi: 10.1038/s41598-022-21249-6 (PMC9633749; doi:10.1038/s41598-022-21249-6)
Supplement: Supplementary file 1 — Supplementary Information. [file 41598_2022_21249_MOESM1_ESM.pdf]

Supplementary information

Increased autophagy leads to decreased apoptosis during β-thalassaemic mouse and patient erythropoiesis

Pornthip Chaichompoo[1,2,*], Ramaneeya Nithipongvanitch[2,*], Wasinee Kheansaard[2,3], Alisa Tubsuwan[2,4], Kanitta Srinoun[2,5], Jim Vadolas[6,7], Suthat Fucharoen[2], Duncan R. Smith[8], Pranee Winichagoon[2], Saovaros Svasti[2, 9,**]

[1]Department of Pathobiology, Faculty of Science, Mahidol University, Bangkok, Thailand; [2]Thalassemia Research Center, Institute of Molecular Biosciences, Mahidol University, Nakhon Pathom, Thailand; [3]Department of Clinical Microscopy, Faculty of Medical Technology, Mahidol University, Bangkok, Thailand; [4]Stem Cell Research Group, Institute of Molecular Biosciences, Mahidol University, Nakhon Pathom, Thailand; [5]Faculty of Medical Technology, Prince of Songkla University, Songkhla, Thailand; [6]Centre for Cancer Research, Hudson Institute of Medical Research, Melbourne, Australia; [7]Department of Molecular and Translational Science, Monash University, Melbourne, Australia; [8]Molecular Pathology Laboratory, Institute of Molecular Biosciences, Mahidol University, Nakhon Pathom, Thailand; and [9]Department of Biochemistry, Faculty of Science, Mahidol University, Bangkok, Thailand

* These authors contributed equally to this work.

****Correspondence:**

Saovaros Svasti, Ph.D.

Thalassemia Research Center, Institute of Molecular Biosciences, Mahidol University, Salaya, Nakhon Pathom 73170 Thailand.

Phone: +662-889-2558; Fax: +662-889-2559; e-mail: saovaros.sva@mahidol.ac.th

Number of supplementary tables: 2

Number of supplementary figures: 9

Supplementary Table

Supplementary Table 1. Lists of monoclonal antibody cocktails for flow cytometric analysis.

Reactivity	Description	Cocktail monoclonal antibodies
Murine	PS-bearing erythroid cells	FITC anti-CD71<br>Biotin anti-TER119/PerCP-Cy5.5 Streptavidin<br>APC annexin V
	Mitochondrial transmembrane potential	$DiOC_6(3)$ (FL1 channel)<br>PE anti-CD71<br>APC anti-TER119
	Activated caspase 8	FITC-IETD-FMK caspase 8<br>PE anti-CD71<br>APC anti-TER119
	Activated caspase 9	FITC anti-CD71<br>Red-LEHD-FMK caspase 9 (FL2 channel)<br>APC anti-TER119
Human	PS-bearing erythroid cells	FITC annexin V<br>Biotin anti-CD71/PerCP-Cy5.5 Streptavidin<br>APC anti-CD235a (GPA)
	Mitochondrial transmembrane potential	FITC anti-CD71<br>TMRE (FL2 channel)<br>PerCP anti-CD45<br>APC anti-CD235a (GPA)
	Activated caspase 3<br>(ICC assay)	FITC anti-CD71<br>PE anti-active caspase 3<br>PerCP anti-CD45<br>APC anti-CD235a (GPA)
	Isotype control for ICC	FITC anti-CD71<br>PE mouse IgG1-PE<br>PerCP anti-CD45<br>APC anti-CD235a (GPA)

APC; Allophycocyanin, $DiOC_6(3)$; 3,3’-dihexyloxacarbocyanine iodide, FITC; fluorescein isothiocyanate, GPA; glycophorin A, ICC; intracellular staining, PE; R-Phycoerythrin, and TMRE; Tetramethylrhodamine ethyl ester.

Supplementary Table 2. Haematological parameters of β-thalassaemia/HbE patients and control subjects.

Description	Control subjects			β-Thalassaemia/HbE patients			Reference range
Number (Male: Female)	3 (2: 1)			6 (2: 4)			
Age (years) (range)	35±10 (28-46)			37±9 (25-45)			
Haemoglobin typing							
HbA (%)	90	±	5				85-90
HbA_2/HbE (%)	3	±	0.2	51	±	8*	2-3
HbF (%)	0.6	±	0.4	41	±	13*	0.8-2
Red blood cell count ($\times 10^6/\mu L$)	4.5	±	0.8	3.2	±	0.6*	4.2-5.4
Haemoglobin (g/dL)	13.6	±	1.7	7.0	±	1.4*	12-18
Haematocrit (%)	40	±	4	24	±	5*	37-52
MCV (fL)	90	±	9	73	±	8*	80-99
MCH (pg)	31	±	3	21	±	3*	27-31
MCHC (g/dL)	34	±	0.6	30	±	4.2	31-35
Red cell distribution width (%)	12.3	±	1.2	23.6	±	0.6*	11.5-14.5
NRBCs (cells/100WBCs)	0	±	0	172	±	326*	None
WBC count ($\times 10^3/\mu L$)	7.0	±	2.3	12.6	±	8.4	4-11
Platelet count ($\times 10^3/\mu L$)	235	±	25	462	±	314	150-450

Data presents in mean±standard deviation (S.D.). MCH; mean corpuscular haemoglobin, MCHC; mean corpuscular haemoglobin concentration, MCV; mean corpuscular volume, NRBCs; nucleated red blood cells and WBC; white blood cells. *Significant different when compared to control subjects at $P < 0.05$.

Supplementary Figures

Wild type mouse

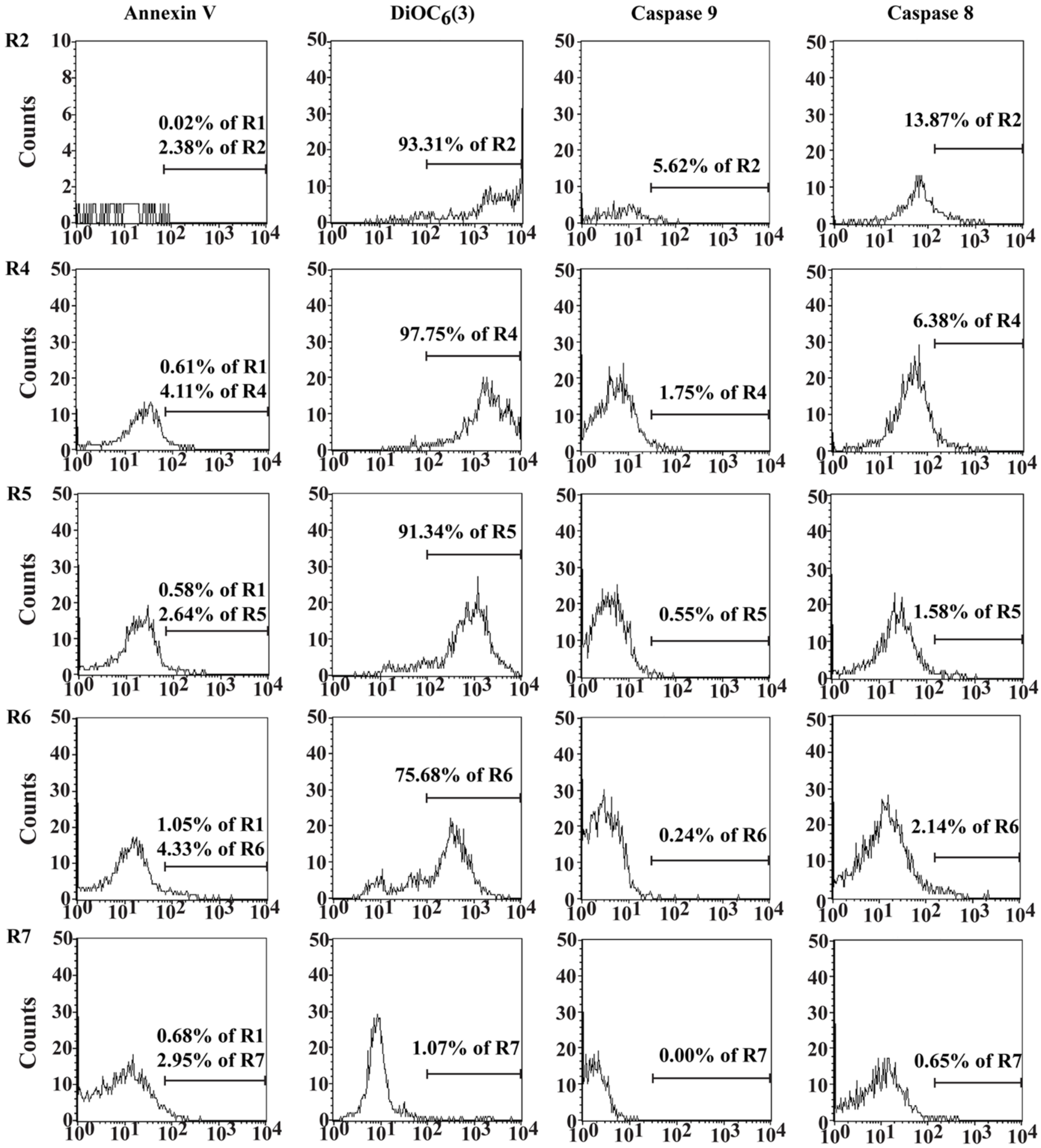

Figure S1. Flow cytometric analysis of apoptosis of different stages of murine bone marrow erythroid cells from wild type mouse (WT). Whole bone marrow from WT was stained with fluorochrome conjugated with monoclonal antibodies specific to CD71 and TER119 to classified stages of erythroid cells from proerythroblasts to mature red blood cells using CD71/TER119/FSC-H as defined by R2, R4-R7 region (Fig. 1A). Then, sample was combined with a third-color fluorescent channel staining including annexin V as phosphatidylserine marker, $DiOC_6(3)$ as mitochondrial transmembrane potential marker, active caspase 9 and active caspase 8 (Supplementary Table 1).

$\beta^{IVS2\text{-}654}$-Thalassaemic mouse

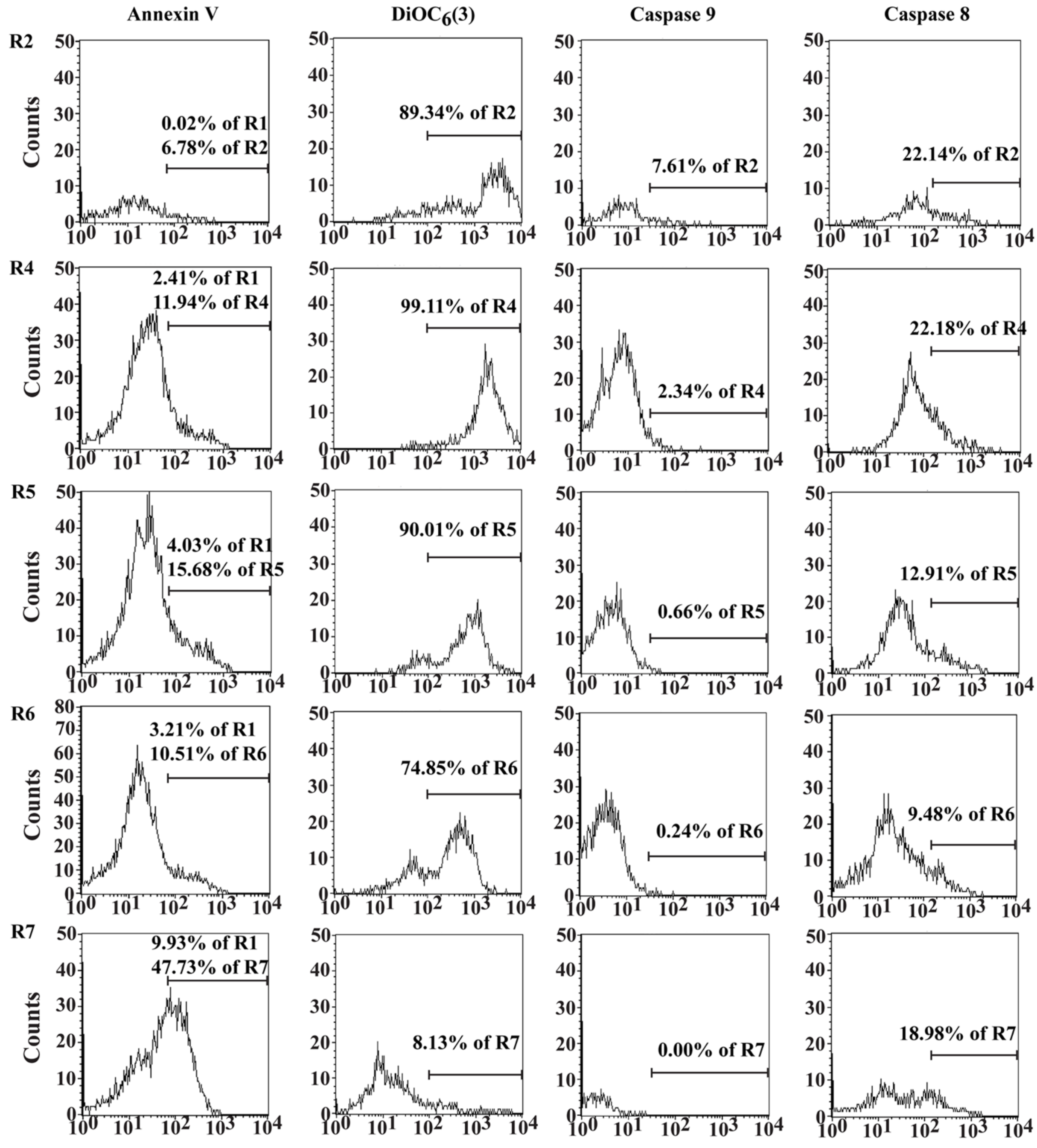

Figure S2. Flow cytometric analysis of apoptosis of different stages of murine bone marrow erythroid cells from $\beta^{IVS2\text{-}654}$-thalassaemic mouse model (654). Whole bone marrow sample was stained with fluorochrome conjugated with monoclonal antibodies specific to CD71 and TER119 to classified stages of erythroid cells from proerythroblasts to mature red blood cells using CD71/TER119/FSC-H as defined by R2, R4-R7 region (Fig. 1A). Then, sample was combined with a third-color fluorescent channel staining including annexin V as phosphatidylserine marker, $DiOC_6(3)$ as mitochondrial transmembrane potential marker, active caspase 9 and active caspase 8 (Supplementary Table 1).

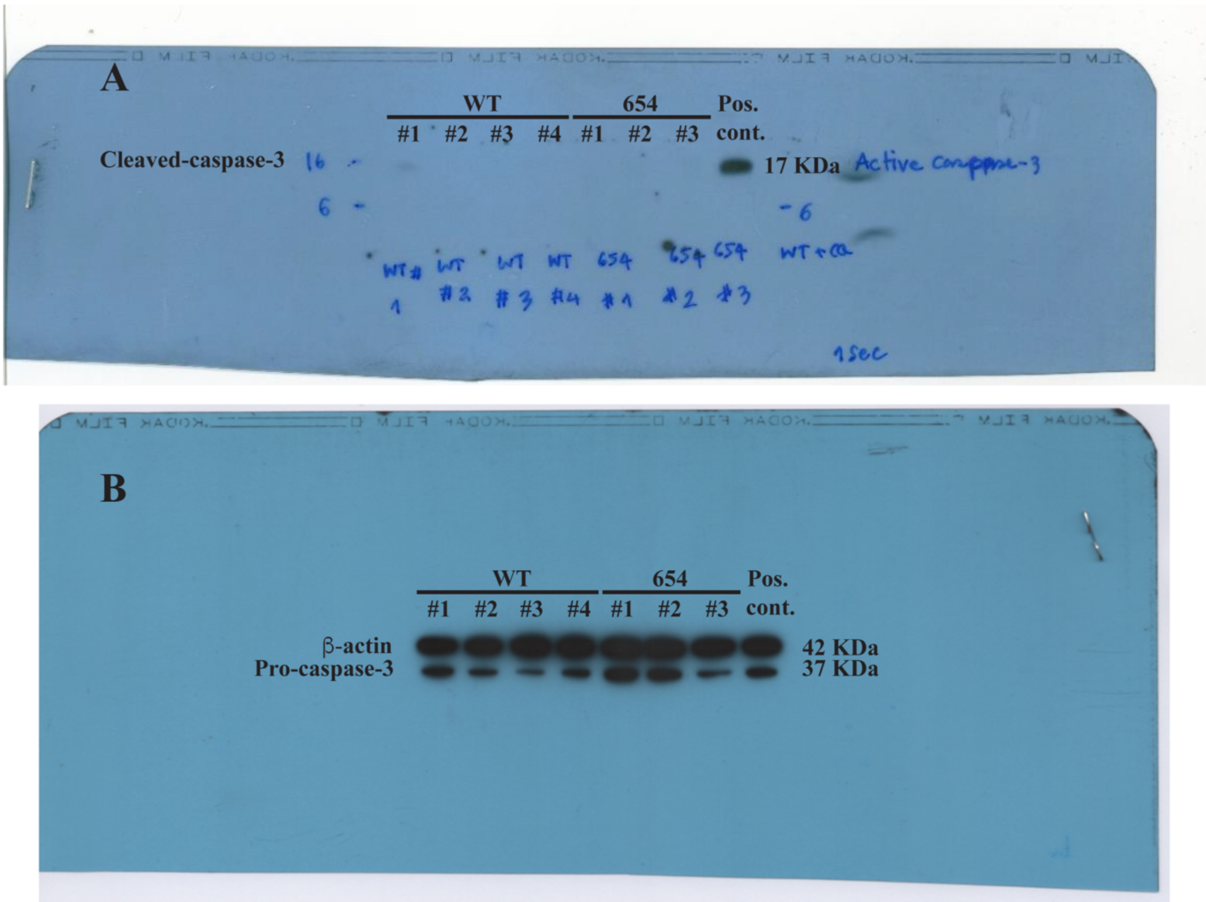

Figure S3. Representative X-ray film of Western blot analysis of caspase 3 in $CD45^{-}CD71^{+}$ bone marrow erythroblasts. Cisplatin-treated $CD45^{-}CD71^{+}$ bone marrow erythroblasts were used as a positive control. The film was exposed for 1 sec. WT; wild type mice, 654; $\beta^{IVS2-654}$-thalassaemic mice.

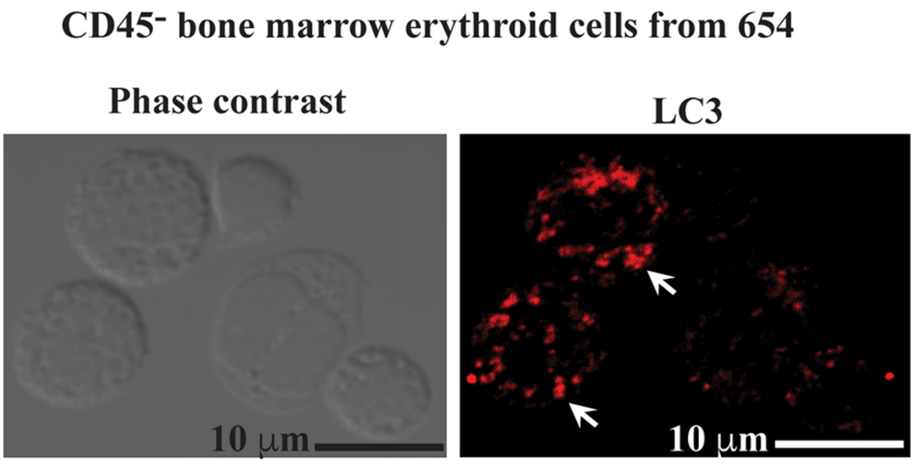

Figure S4. Calculation of percentages of autophagosome positive in murine bone marrow erythroid cells. $CD45^-$ bone marrow erythroid cells from β-thalassaemic mice were stained with fluorochrome conjugated with specific antibody to LC3 for analysis of the punctate LC3 expression, classified as autophagosome using confocal microscope. The percentages of $autophagosome^+$ cells were counted and calculated. Total erythroid cells were counted from phase contrast. Arrows are identified autophagosomes.

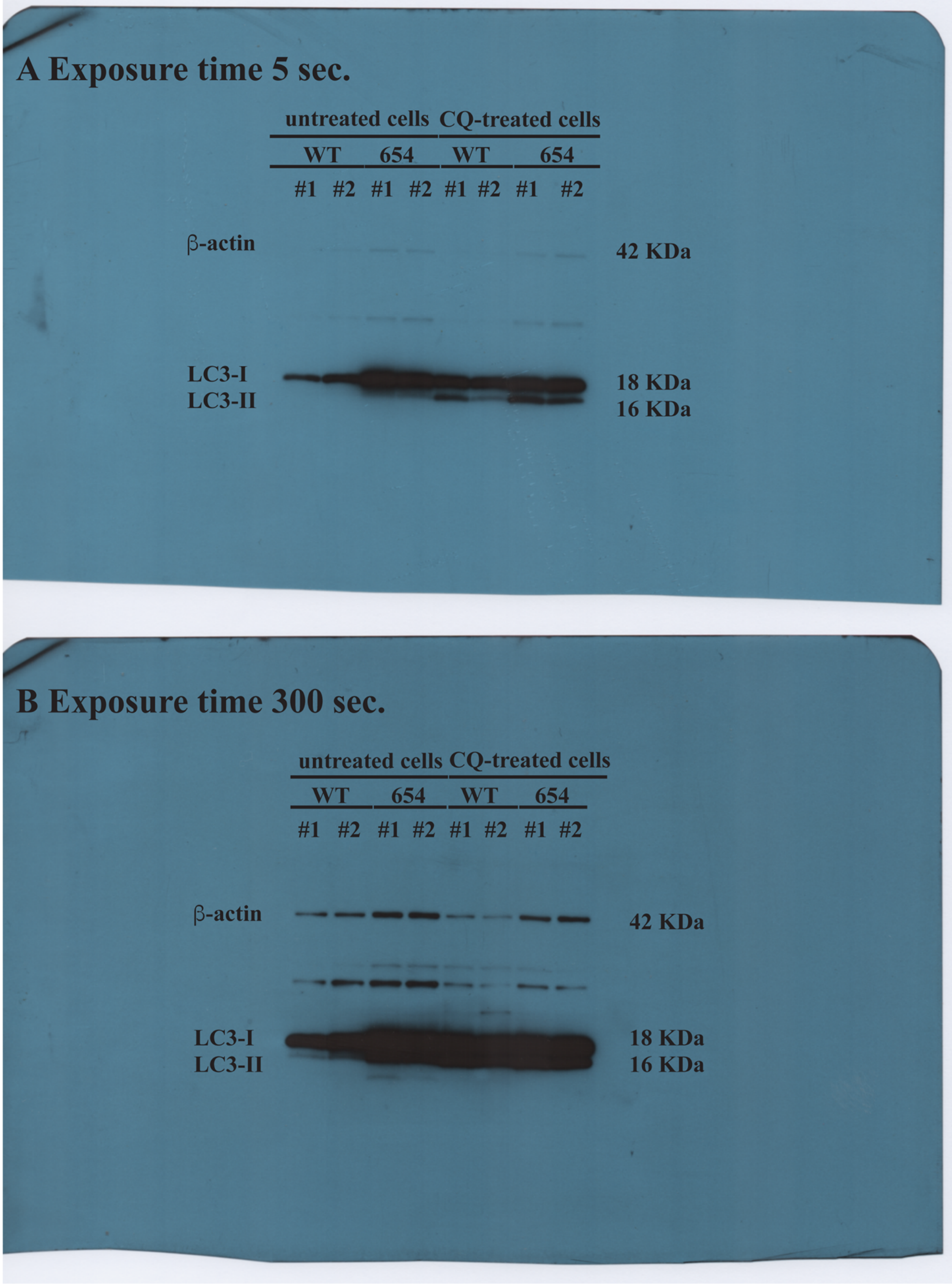

Figure S5. Representative X-ray film of Western blot analysis of LC3-I and LC3-II in $CD45^-$ $CD71^+$ bone marrow erythroblasts treated with 100 μM chloroquine (CQ). The film was exposed for (A) 5 and (B) 300 sec. WT; wild type mice, 654; $\beta^{IVS2\text{-}654}$-thalassaemic mice.

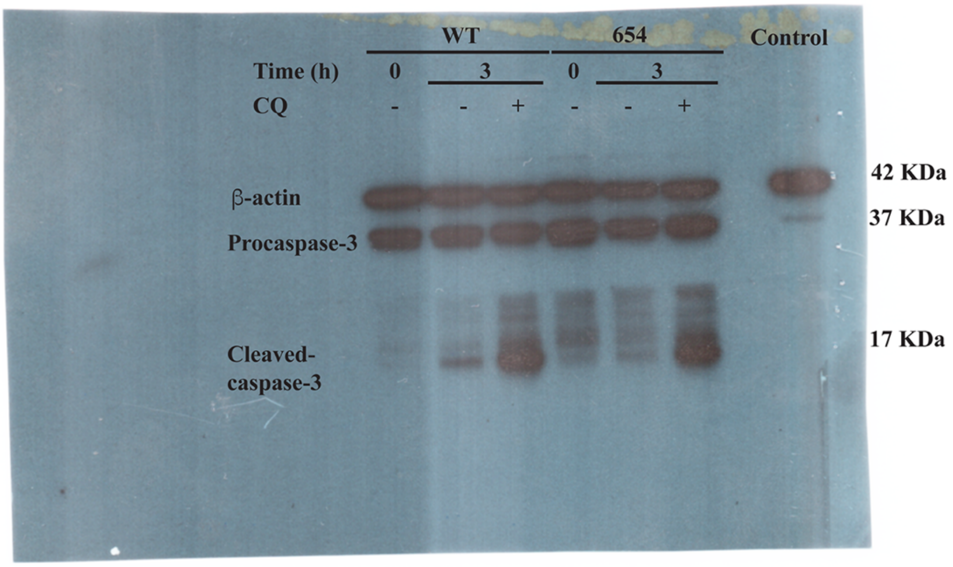

Figure S6. Representative X-ray film of Western blot analysis of caspase 3 in $CD45^{-}CD71^{+}$ bone marrow erythroblasts treated with 100 μM chloroquine (CQ). The film was exposed for 1 sec. WT; wild type mice, 654; $\beta^{IVS2\text{-}654}$-thalassaemic mice.

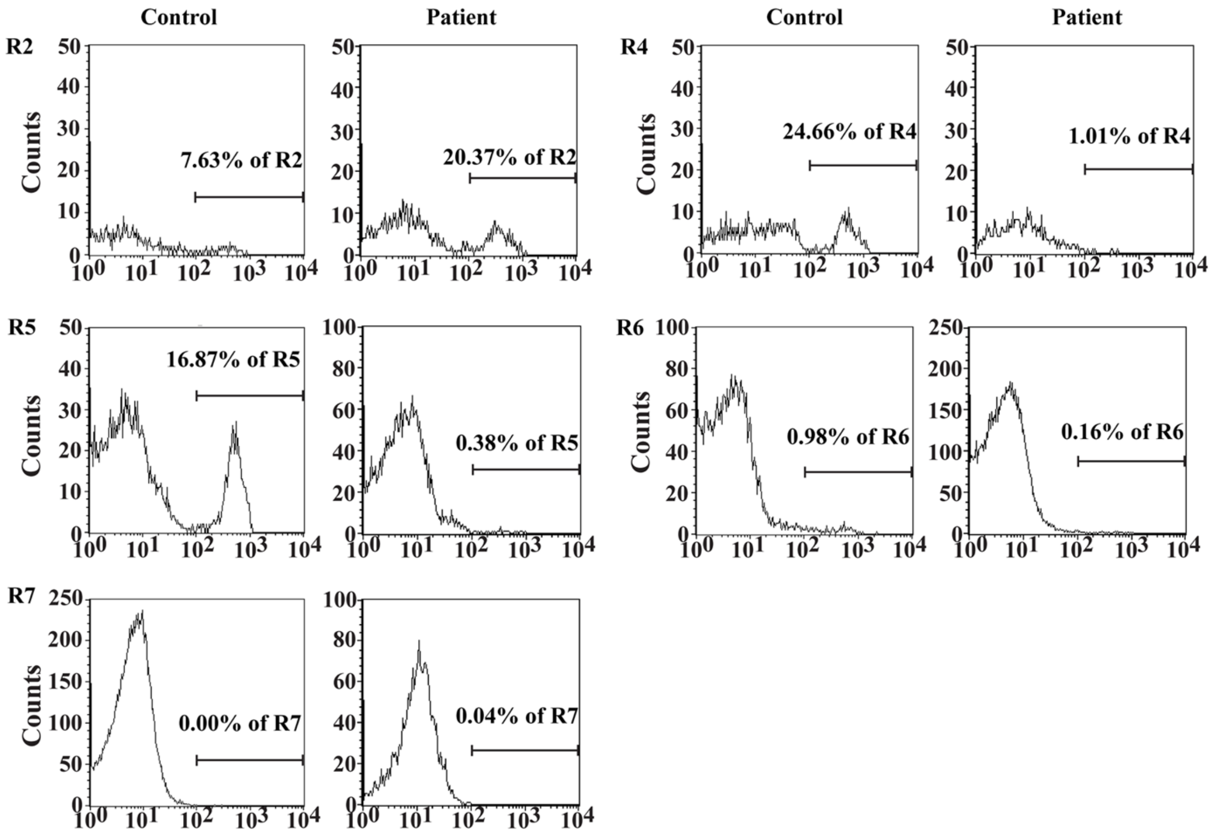

Figure S7. Flow cytometric analysis of activated caspase 3 in each subpopulation of erythroid cells from bone marrow of control subject and β-thalassaemia/HbE patient. Whole bone marrow sample was fixed and permed with cold-cytoFix/Perm solution and stained with FITC conjugated anti-CD71, APC conjugated anti-GPA, PerCP conjugated anti-CD45 and PE conjugated anti-activated caspase 3 using the intracellular staining assay. The stages of erythroid cells were classified into R2, R4-R7 region (Fig. 4A). Then, each erythroid subpopulation was analysed the percentages of activated caspase 3 using CellQuest Pro^{TM} software.

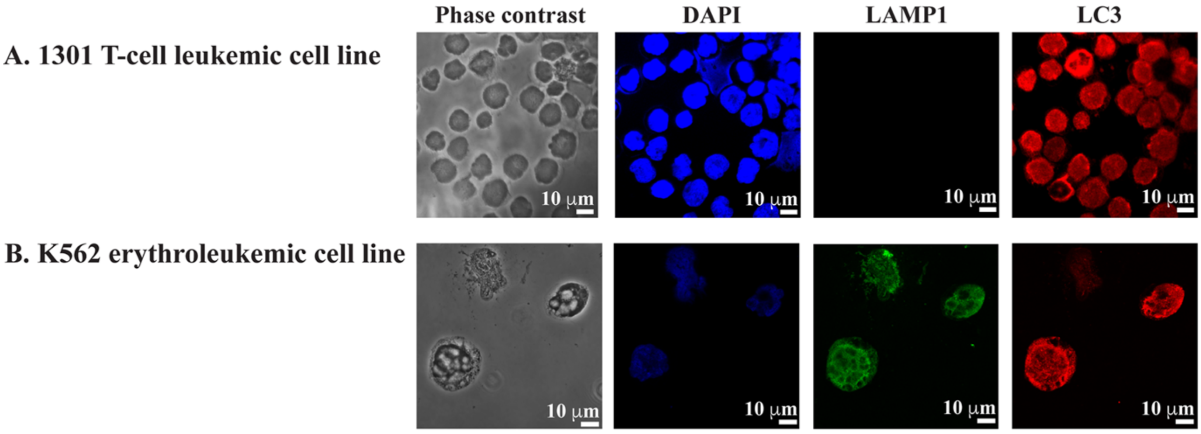

Figure S8. LAMP1 negative detection in lymphocytes by using anti-LAMP1 monoclonal antibody, clone 1D4B (BioLegend). (A) The 1301 T-cell leukemic cell line and (B) the K562 erythroleukemic cell line were cultured in serum free media for 4 hr as starvation induced autophagy. Cells (5×10^4) were harvested and fixed on the slide to stained with DAPI (blue), FITC conjugated anti-LAMP1 (green) and Cy5 conjugated goat anti-rabbit/rabbit anti-LC3 (red). Illustration was captured using an Olympus FluoView 1000 confocal microscope (Olympus, Tokyo, Japan) at 60× objective lens.

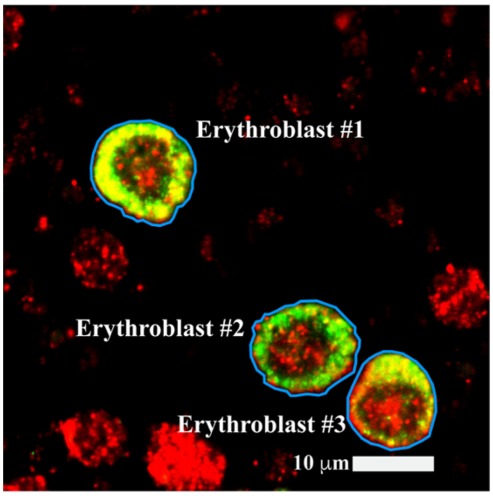

Erythroblast no.	Pearson's correlation coefficient	Interpretation
#1	0.67794	positive autophagy
#2	0.17663	negative autophagy
#3	0.43767	negative autophagy

Figure S9. Identification of autophagic cells using a confocal microscope. The $CD45^-$ human bone marrow erythroblasts were stained with FITC conjugated anti-LAMP1 (green) and Cy5 conjugated goat anti-rabbit/rabbit anti-LC3 (red) to analyse autophagy. The erythroblasts were identified using morphology by phase contrast analysis. Co-localization between LAMP-1 and LC3 presents in yellow and was measured by Pearson's correlation coefficients by Olympus FluoView software, an Olympus confocal laser scanning microscope FV10i-DOC (Olympus Corporation, Tokyo, Japan). Illustration was captured using a 60× objective lens. The individual $CD45^-$ bone marrow erythroblasts were drawn and analysed for Pearson's correlation coefficients for the interpretation of positive autophagic cells by using the cut-off at ≥0.5. In this image, there are 3 erythroblast cells as making cell number #1, #2 and #3, the erythroblast number #1 was presented the Pearson's correlation coefficients 0.67794 as identifying as positive autophagic cell while another 2 erythroblasts were negative autophagy as the Pearson's correlation coefficients < 0.5. The non-erythroblasts, classified by morphology using phase contrast, were not counted.
